# Markers of low field NMR relaxation features of tissues

**DOI:** 10.1038/s41598-024-74055-7

**Published:** 2024-10-22

**Authors:** Karol Kołodziejski, Elzbieta Masiewicz, Amnah Alamri, Vasileios Zampetoulas, Leslie Samuel, Graeme Murray, David J. Lurie, Lionel M. Broche, Danuta Kruk

**Affiliations:** 1 Department of Physics and Biophysics, University of Warmia & Mazury in Olsztyn, Oczapowskiego 4, 10-719 Olsztyn, Poland; 2 School of Medicine, Medical Sciences and Nutrition, Biomedical Physics, University of Aberdeen, Foresterhill, Aberdeen, AB25 2ZD UK; 3 Department of Oncology, Aberdeen Royal Infirmary, Aberdeen, UK; 4 Department of Radiologic Sciences, Faculty of Applied Medical Sciences, King Abdulaziz University, Jeddah, Saudi Arabia

**Keywords:** Biophysics, Biomarkers, Tumour biomarkers, Biological physics

## Abstract

This work presents an approach to exploiting Nuclear Magnetic Resonance (NMR) relaxometry data (^1^H spin-lattice relaxation rates covering the frequency range from below 1 kHz to 10 MHz) for the purpose of differentiating between pathological and reference tissues. Characteristic quantities (markers) that can be obtained in a straightforward manner, not resorting to an advanced analysis of ^1^H spin-lattice relaxation data, have been identified and compared for pathological and reference colon tissues. Moreover, the relaxation data have been parametrised in terms of Lorentzian spectral densities and the possibility of using the obtained dipolar relaxation constants and correlation times as biomarkers to assess the state of tissues has been discussed. It has also been demonstrated that the relaxation data for the reference and the pathological tissues can be attributed to two groups (for each case). The studies are a step towards exploiting the potential of NMR relaxometry for characterisation of pathological changes in tissues.

**Supplementary Information:**

The online version contains supplementary material available at 10.1038/s41598-024-74055-7.

## Introduction

Worldwide, colorectal cancer (CRC) ranks as the third most common kind of cancer and the third-leading cause of cancer-related death, with nearly one million annual fatalities^1,2^. The incidence and mortality rates of colorectal cancer increased significantly from 1990 to 2019, with incident cases and deaths more than doubling^3^. Early detection of CRC, risk stratification, prevention, and treatment principles are profoundly impacted by the biological complexity of CRC, which necessitates a multidimensional comprehension and development of personalised care and early detection biomarkers^4^. In human cells, the transition from a normal to a malignant state is the result of intricate interactions between genetic, environmental factors and cellular dynamics. Research has shown that changes in the dynamical activity of intracellular water play a role in differentiating the cellular state between healthy and cancer cells. This understanding offers crucial insights into the changes in dynamics underlying carcinogenesis and tumour metastasis^5,6^. Moreover, the water permeability of CRC cell membranes differs from that of healthy cells, possibly due to mutations in aquaporin channels. This alteration affects the uptake of nutrients and the resistance to drugs. Aquaporins (AQPs) are a group of transmembrane water channel proteins and are vital in maintaining water homeostasis^7^. The aforementioned biomarkers were investigated previously in several pathological conditions using field cycling nuclear magnetic resonance (FC-NMR) and show promising potential in the assessment of tissue remodelling non-invasively and without using contrast media^8–11^.

The Nuclear Magnetic Resonance (NMR) phenomenon is the foundation of Magnetic Resonance Imaging (MRI) that revolutionised medical diagnostics. The essence of MRI lies in differences between parameters referred to as relaxation times (spin-lattice and spin-spin relaxation times, $\:{T}_{1}$ and $\:{T}_{2}$, respectively – their reciprocal values are referred to as relaxation rates: $\:{R}_{1}=1/{T}_{1}$ and $\:{R}_{2}=1/{T}_{2}$). MRI is usually performed at high magnetic fields to maintain high spatial resolution. This undeniably critical factor in medical diagnostics implies that the observed differences in the relaxation rates between healthy and pathological tissues are mainly associated with changes in the dynamical properties of water molecules in tissues. This is related to the fundamentals of NMR relaxation – the dominating relaxation contribution is associated with dynamical processes occurring on a timescale matching the reciprocal resonance frequency (that is proportional to the magnetic field)^11–14^. Consequently, at high magnetic fields (the magnetic field of 1T corresponds to ^1^H resonance frequency of about 42 MHz) the relaxation rates are dominated by contributions associated with fast dynamics of small molecules. In this context, it is natural to wonder whether relaxation studies at lower magnetic fields could reveal pathological changes in tissues that cannot be detected at high magnetic fields.

Fast Field Cycling technology enables relaxation experiments to be performed over a very broad range of resonance frequencies (magnetic fields), from below 1 kHz to tens of MHz (referring to ^1^H resonance frequency). Consequently, these kinds of studies, referred to as NMR relaxometry, possess the unique potential to probe dynamical processes occurring on the time scale from milliseconds to nanoseconds in a single experiment. On top of that, the shape of the frequency dependencies of spin-lattice relaxation rates (referred to as NMR dispersion curves) reflect the mechanism of the motion – in this way one can not only distinguish between translational and rotational dynamics^15–20^, but also reveal anisotropy of the motion^21–24^. Moreover, at lower frequencies (around 1 MHz) one can detect specific relaxation features, referred to as Quadrupole Relaxation Enhancement (QRE)^25,26^ associated with the presence of nuclei possessing quadrupole moments – for organic molecules typically ^14^N. The QRE effects manifest themselves as frequency-specific relaxation maxima that appear only in the case of slow molecular motion^25–27^, an example of which is immobilised proteins in tissues^26–31^.

These unique advantages of NMR relaxometry have been exploited to reveal dynamical properties of molecular and ionic systems of various complexity, from “simple” liquids^32–34^ via macromolecules, such as proteins^22,24,27,31,35–42^ or polymers^43,44^ to tissues^8,10,11,45–53^. As far as tissues are concerned, one should turn attention to the studies of relaxation effects in osteoarthritic articular cartilage^45–47^, breast tissues^48–50^, muscle tissues (sarcoma)^8^ and glioma^11,51^. One should also note the insight provided by NMR relaxometry into water dynamics in intracellular and extracellular matrices^10,48,52,53^. Although NMR relaxometry studies of tissues are very limited, the potential of this approach has led to the construction of a prototype of an MRI scanner that uses magnetic field cycling to operate at different magnetic fields^9^.

In this work, we continue the promising studies aiming at introducing a set of parameters that can be straightforwardly obtained from ^1^H spin-lattice relaxometry data for tissues, not requiring an advanced analysis. Although a quantitative analysis of NMR relaxometry data for tissues, based on specific models of motion, can be a rich source of information about the dynamics of different molecular fractions in tissues, in this paper we do not go in that direction. Our goal is to reveal relaxation features (biomarkers) that can directly be compared for healthy (reference) and pathological tissues for the purpose of assessing the state of the tissue. The indicated biomarkers are compared for colon tissues. To avoid any misunderstanding, at present the results of this work are far from serving diagnostic purposes; nevertheless, they are a step in this direction. In the first part of the work, we focus on relaxation markers that do not require any data processing and can be considered separately – i.e. without a comparison with counterpart data for a reference or for a pathological tissue. Next, we reproduce the data in terms of fundamental (and the simplest) relaxation expressions. These serve two purposes: the first is to use the theoretical curves to determine other “simple” markers not affected by scatter of the experimental data; the second purpose is to compare the quantities obtained from the parametrisation of the data, as they also may be used (to some extent) to distinguish between reference and pathological tissues. Finally, the relaxation data for the reference and the pathological tissues have been grouped with respect to the shape of the frequency dependencies of the relaxation rates (not their arbitrary values). As already pointed out, this approach is intended to be a step toward revealing changes in the molecular dynamics and arrangement caused by pathological changes in tissue with the aim of turning attention to the diagnostic potential of NMR relaxometry.

## Results

### Comparison of slopes

**Fig. 1:**
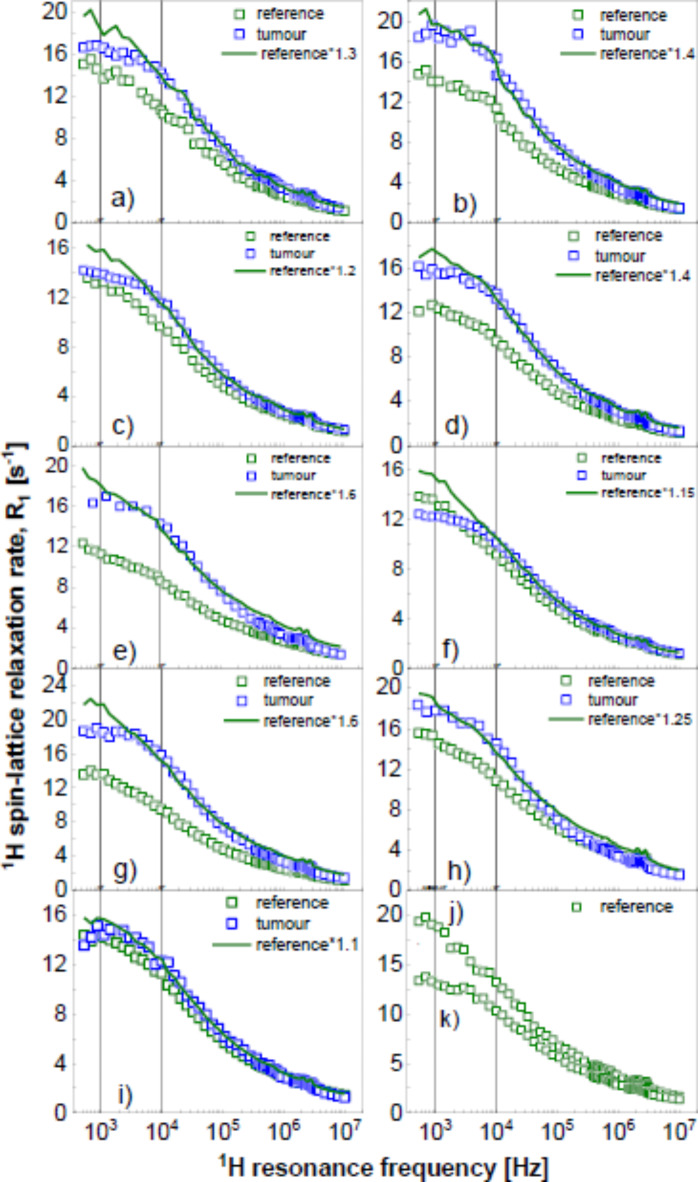
^1^H spin-lattice relaxation rates for pathological and reference (background) colon tissues. Solid lines – re-scaled relaxation rates for the reference samples. Vertical lines indicate the frequency range used for calculating the $\:\xi\:$ parameter.

As already pointed out, the purpose of relaxation markers is to quickly assess the state of tissues without requiring a full, quantitative analysis of the ^1^H spin-lattice relaxation data. In other words, the markers are meant as relaxation features (described by quantitative parameters) that are seen “at the first glance”. To illustrate this concept, a data set including nine examples of ^1^H spin-lattice relaxation data for pathological and reference (background) colon tissues (kept in formalin) have been selected. The data are shown in Fig. 1. The data (Fig. 1 a to i) are complemented by two more cases (Fig. 1 j and k) illustrating data for reference tissues only. In the first nine cases (a to i), the ^1^H spin-lattice relaxation rates for the reference tissues have been multiplied by a factor (indicated in the Figure legend) chosen in such a way so the data for the reference and the pathological tissues overlap in the high frequency range. This re-scaling has been applied to better visualise differences in the shapes of the relaxation dispersion profiles (spin-lattice relaxation rates versus the resonance frequency). As one can see from Fig. 1, after the re-scaling, the relaxation rates mostly differ in the low frequency range. This observation can be exploited to define the first relaxation marker – one can look at the relative differences in the relaxation rates in the low frequency range encompassing one order of magnitude. Thus, the parameters associated with this marker can be defined as: $\:\xi\:=\left|\frac{{R}_{1}\left({\nu\:}_{1}\right)-{R}_{1}\left({\nu\:}_{2}\right)}{{R}_{1}\left({\nu\:}_{2}\right)}\right|$, where $\:{\nu\:}_{1}$ and $\:{\nu\:}_{2}$ denote the resonance frequencies determining the chosen frequency range ($\:{\nu\:}_{1}$ refers to the lower frequency), while $\:{R}_{1}\left({\nu\:}_{1}\right)$ and $\:{R}_{1}\left({\nu\:}_{2}\right)$ are the corresponding relaxation rates at those frequencies. The parameter $\:\xi\:$ reflects, to some extent, the “steepness” of the frequency dependencies of the relaxation rates in the interval $\:\left[{\nu\:}_{1},\:{\nu\:}_{2}\right]$, but it is (in principle) independent of the arbitrary values of the relaxation rates. The frequency interval can be chosen as one wishes, but before doing that one should be aware in which range the differences in the $\:\xi\:$ parameter between the relaxation data for pathological and reference tissues are most likely to occur. In the present case the frequencies are about $\:{\nu\:}_{1}$ = 1 kHz and $\:{\nu\:}_{2}$ = 10 kHz (that means a very small frequency range). The values of the parameter $\:\xi\:$ are collected in Table 1.

**Table 1:** $\:\xi\:$ values (in %) obtained for the relaxation data shown in Fig. 1 in the range from 1 kHz to 10 kHz. Note that $\:\xi\:$ is unitless.

Case	Pathological	Reference	Pathological	Reference
	Raw data		Fitted data	
(a)	16.8	27.6	16.8	33.8
(b)	12.6	23.6	26.7	23.3
(c)	13.9	37.2	20.2	36.2
(d)	16.9	31.3	17.6	28.8
(e)	18.8	30.7	14.3	30.8
(f)	20.8	42.9	20.6	43.2
(g)	16.8	43.8	17.1	43.3
(h)	29.2	33.8	28.0	33.8
(i)	17.3	25.6	18.3	23.6
(j)	–	42.1	–	42.0
(k)	–	25.5	–	25.4
Average	18.1	33.1	20.0	34.1
Standard deviation	4.5	6.7	4.6	7.2

Looking at the obtained averaged values and their standard deviations, one can see that relaxation data for the pathological tissues exhibit a lower steepness compared to those for the reference samples. The slope values for the pathological tissues vary from 13.6 to 22.6%, while for the reference tissues they range from 28.1 to 41.5 for the raw data. In the case of the pathological tissues, the slope values range from 15.5 to 24.3, and for the fitted data they range from 27.0 to 42.2.

Although the purpose of the markers is to assess the differences in the relaxation features as fast as possible, in case of scattered data, it is useful to interpolate (spline) the data. This has been done in Fig. A1 shown in the Supplementary Appendix. Table 1 includes the values of the $\:\xi\:$ parameter obtained from the interpolated data for comparison. The conclusion remains unchanged. It is of interest to extend the frequency range, including three orders of magnitude from 10 kHz to 10 MHz. In this way, the shape of the frequency dependence of the spin-lattice relaxation rate is lost, but the difference in the $\:\xi\:$ parameter reflects the changes in the relaxation rates resulting from dynamical processes occurring on much different time scales. The values of the $\:\xi\:$ parameter for the frequency range from 10 kHz to 10 MHz for the raw data and the smoothed ones are collected in Table 2.

**Table 2:** $\:\xi\:$ values (in %) obtained for the relaxation data shown in Fig. 1 in the range from 10 kHz to 10 MHz.

Case	Pathological	Reference	Pathological	Reference
	Raw data		Fitted data	
(a)	1040	808	857	808
(b)	974	649	974	649
(c)	924	752	925	752
(d)	1040	710	936	618
(e)	983	553	966	543
(f)	816	727	816	727
(g)	1250	709	1010	709
(h)	775	582	986	578
(i)	778	709	770	709
(j)	–	866	–	866
(k)	–	653	–	652
Average	955	696	916	684
Standard deviation	146	88	74	92

The slope for the pathological tissues ranges between 809 and 1100 and for the reference ones between 608 and 783 for the raw data and between 838 and 994 for the pathological tissues and between 591 and 777 for the interpolated data.

### Parametrisation of the data

The frequency dependencies of the spin lattice relaxation rates, $\:{R}_{1}\left(\omega\:\right)$ ($\:\omega\:$ denotes the resonance frequency in angular frequency units), can be reproduced based on the well-known expressions linking the relaxation rates with the time scale of the molecular motion. According to the relaxation theory, the spin-lattice relaxation rate can be expressed as^14^:

3 $$\:{R}_{1}\left(\omega\:\right)=C\left[\frac{{\tau\:}_{c}}{1+{\left(\omega\:{\tau\:}_{c}\right)}^{2}}+\frac{{4\tau\:}_{c}}{1+{\left(2\omega\:{\tau\:}_{c}\right)}^{2}}\right]$$

where the parameter $\:{\tau\:}_{c}$ is referred to as a correlation time and describes the time scale of the molecular motion associated with the relaxation process, while $\:C$ denotes the corresponding dipolar relaxation constant, reflecting the amplitude of the dipole–dipole interactions. As the experiments have been carried out in a frequency range encompassing more than three orders of magnitude, the relaxation data can be decomposed into three contributions, according to the equation:

4 $$\:{R}_{1}\left(\omega\:\right)={C}_{s}\left[\frac{{\tau\:}_{s}}{1+{\left(\omega\:{\tau\:}_{s}\right)}^{2}}+\frac{4{\tau\:}_{s}}{1+{\left(2\omega\:{\tau\:}_{s}\right)}^{2}}\right]+{C}_{i}\left[\frac{{\tau\:}_{i}}{1+{\left(\omega\:{\tau\:}_{i}\right)}^{2}}+\frac{4{\tau\:}_{i}}{1+{\left(2\omega\:{\tau\:}_{i}\right)}^{2}}\right]+{C}_{f}\left[\frac{{\tau\:}_{f}}{1+{\left(\omega\:{\tau\:}_{f}\right)}^{2}}+\frac{4{\tau\:}_{f}}{1+{\left(2\omega\:{\tau\:}_{f}\right)}^{2}}\right]+A$$

The contributions include the indices “$\:s$”, “$\:i$” and “$\:f$” originating from “slow”, “intermediate” and “fast” dynamical processes. This terminology is associated with the values of the correlation times that fulfil the condition: $\:{\tau\:}_{s}>{\tau\:}_{i}>{\tau\:}_{f}$; $\:{C}_{s}$, $\:{C}_{i}$ and $\:{C}_{f}$ denote the corresponding dipolar relaxation constants. The frequency independent term, $\:A$, describes a relaxation contribution originating from dynamical processes that are too fast to lead to changes of the relaxation rates with frequency (the correlation times fulfil the condition $\:\omega\:{\tau\:}_{c}\ll\:1$ in the covered frequency range). The data were fitted in terms of Eq. 4 and the obtained parameters are collected in Table 3. We propose potential interpretations of these contributions in the Discussion, but the histology analyses did not provide sufficient data to validate one and additional work is needed to clarify this.

The data also showed weakly pronounced maxima in the frequency range around 1 MHz. The maxima represent Quadrupole Relaxation Enhancement (QRE) effects and are often referred to as quadrupole peaks. These are well known signals in biological T1 relaxometry that are due to cross-relaxation between water protons and ^14^N from proteins, and have been studied extensively in the literature^15,16^. Because of their low amplitude, we decided to omit them in the analysis.

**Table 3:** Parameters obtained from fitting the relaxation data in terms of Eq. 4.

Case	$\:{C}_{s}$ [10^5^ Hz^2^]	$\:{\tau\:}_{s}$ [µs]	$\:{C}_{i}$ [10^6^ Hz^2^]	$\:{\tau\:}_{i}$ [µs]	$\:{C}_{f}$ [10^7^ Hz^2^]	$\:{\tau\:}_{f}$ [µs]	$\:A$ [s^−1^]
(a) Tumour	2.12 ± 0.10	66.00 ± 2.30	1.72 ± 0.88	0.67 ± 0.15	0.09 ± 0.01	4.40 ± 1.30	1.31 ± 0.23
(a) Reference	0.76 ± 0.18	15.00 ± 4.90	0.98 ± 0.09	1.14 ± 0.08	0.66 ± 0.01	6.50 ± 0.62	1.23 ± 0.11
(b) Tumour	3.62 ± 0.27	5.50 ± 0.43	2.13 ± 0.13	0.49 ± 0.10	1.32 ± 0.19	4.40 ± 1.30	1.41 ± 0.24
(b) Reference	0.68 ± 0.17	18.00 ± 8.00	0.98 ± 0.09	1.30 ± 0.11	0.63 ± 0.02	6.50 ± 0.62	1.22 ± 0.13
(c) Tumour	2.32 ± 0.16	5.70 ± 0.29	1.53 ± 0.75	0.61 ± 0.05	0.89 ± 0.09	3.80 ± 0.37	1.24 ± 0.13
(c) Reference	0.76 ± 0.07	15.00 ± 1.40	0.85 ± 0.04	1.10 ± 0.12	0.72 ± 0.06	4.90 ± 0.74	1.14 ± 0.12
(d) Tumour	2.31 ± 0.20	6.10 ± 0.27	1.52 ± 0.10	0.69 ± 0.06	0.99 ± 0.13	4.70 ± 0.43	1.22 ± 0.14
(d) Reference	0.67 ± 0.10	15.00 ± 6.60	0.77 ± 0.05	1.20 ± 0.09	0.74 ± 0.01	5.00 ± 0.49	1.11 ± 0.13
(e) Tumour	2.14 ± 0.30	6.80 ± 0.28	2.34 ± 0.14	0.58 ± 0.06	1.41 ± 0.19	2.8 ± 0.39	1.24 ± 0.21
(e) Reference	0.21 ± 0.20	32.00 ± 8.20	0.47 ± 0.03	2.10 ± 0.23	0.53 ± 0.04	8.40 ± 1.30	1.53 ± 0.14
(f) Tumour	1.14 ± 0.10	8.50 ± 0.45	0.87 ± 0.05	0.99 ± 0.06	0.65 ± 0.06	6.70 ± 0.43	1.34 ± 0.13
(f) Reference	0.53 ± 0.33	23.00 ± 1.70	0.82 ± 0.03	1.20 ± 0.12	0.66 ± 0.06	5.40 ± 0.89	1.12 ± 0.14
(g) Tumour	3.63 ± 0.25	5.40 ± 0.24	1.92 ± 0.01	0.56 ± 0.06	1.24 ± 0.15	4.00 ± 0.45	1.33 ± 0.14
(g) Reference	0.42 ± 0.45	27.00 ± 2.20	0.66 ± 0.04	1.60 ± 0.17	0.61 ± 0.05	6.5 ± 0.82	1.34 ± 0.12
(h) Tumour	2.56 ± 0.11	6.90 ± 0.34	1.73 ± 0.02	0.63 ± 0.06	0.96 ± 0.09	4.30 ± 0.33	1.53 ± 0.24
(h) Reference	0.17 ± 0.14	50.00 ± 5.60	0.44 ± 0.04	3.10 ± 0.13	0.74 ± 0.06	8.60 ± 1.60	1.94 ± 0.13
(i) Tumour	2.1 ± 0.11	6.10 ± 0.29	1.33 ± 0.03	0.71 ± 0.06	0.86 ± 0.01	5.40 ± 0.56	1.32 ± 0.13
(i) Reference	0.71 ± 0.13	14.00 ± 6.70	0.79 ± 0.05	1.40 ± 0.14	0.66 ± 0.05	7.30 ± 0.77	1.53 ± 0.14
(j) Reference	0.37 ± 0.43	35.00 ± 4.90	0.65 ± 0.08	2.40 ± 0.33	0.78 ± 0.06	9.00 ± 0.81	1.93 ± 0.13
(k) reference	0.35 ± 0.11	24.00 ± 5.30	0.64 ± 0.09	1.70 ± 0.4	0.78 ± 0.07	6.70 ± 0.79	1.44 ± 0.12
Average tumour	2.45 ± 0.08	0.64 ± 0.54	1.63 ± 0.42	0.66 ± 0.03	1.13 ± 0.06	4.40 ± 0.37	1.33 ± 0.05
Average reference	0.51 ± 0.10	24.00 ± 2.30	0.71 ± 0.02	1.40 ± 0.06	0.68 ± 0.02	6.80 ± 0.24	1.44 ± 0.08

The most significant differences between the pathological and reference tissues are observed for the parameters characterising the slow dynamics, $\:{\tau\:}_{s}$ and $\:{C}_{s}$, while the parameters associated with the fast dynamics, $\:{\tau\:}_{i}$ and $\:{C}_{i}$, are comparable. To calculate errors for averaged values, the average of error for each parameter for each group was calculated.

**Fig. 2:**
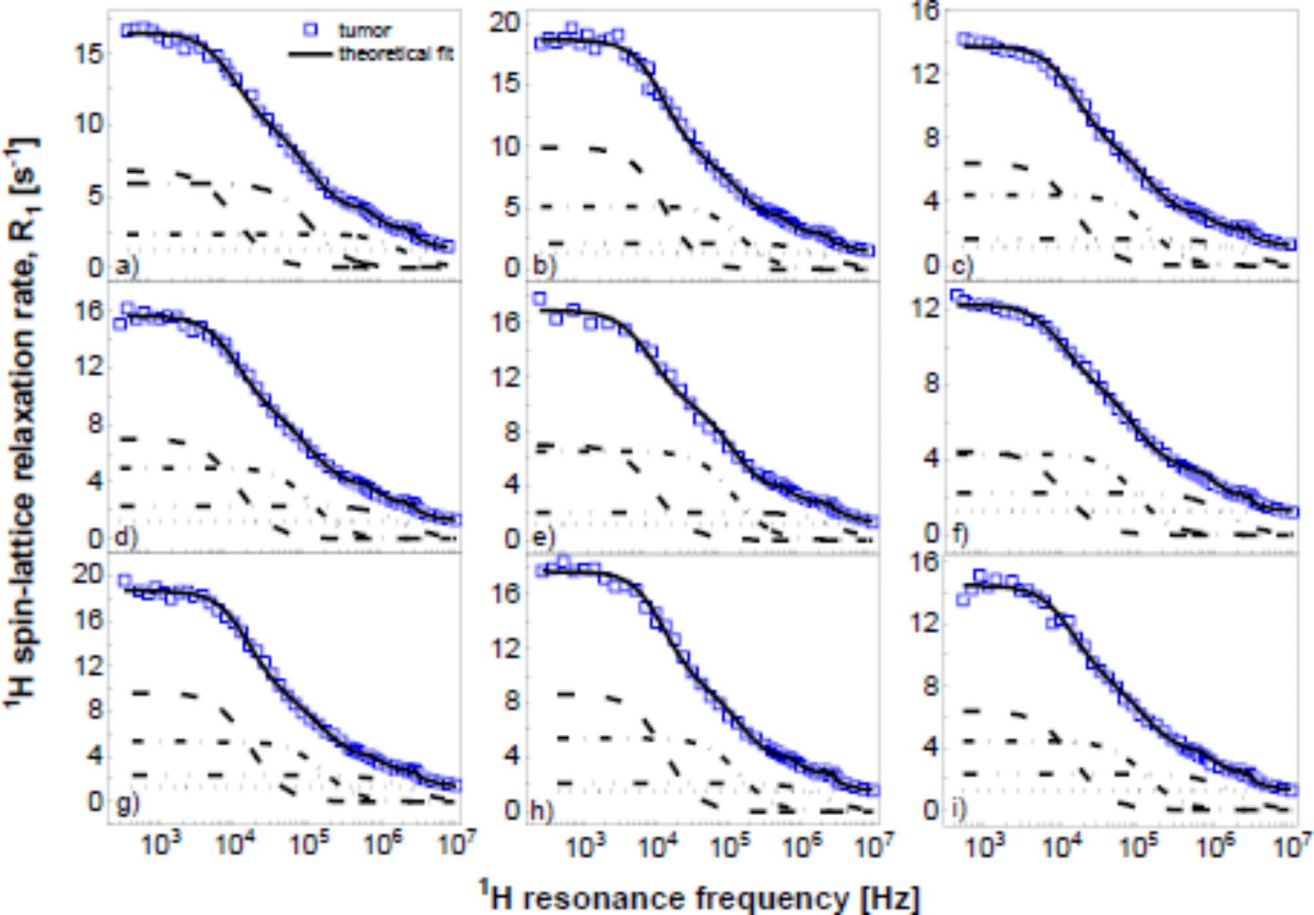
^1^H spin-lattice relaxation rates from pathological colon tissues (blue squares) fitted using Eq. 4 (solid lines). The fit has been decomposed into the relaxation contributions associated with slow (dashed lines), intermediate (dashed-dotted) and fast (dashed-dotted-dotted) lines; the frequency independent term, $\:A$, is represented by dotted lines.

Figure 2 shows the decomposition of the relaxation data into the individual contributions for the pathological samples.

Analogous decomposition of the data for the reference samples is shown in Fig. A2 (Supplementary Appendix).

### Relaxation rates in the derivative representation

**Fig. 3:**
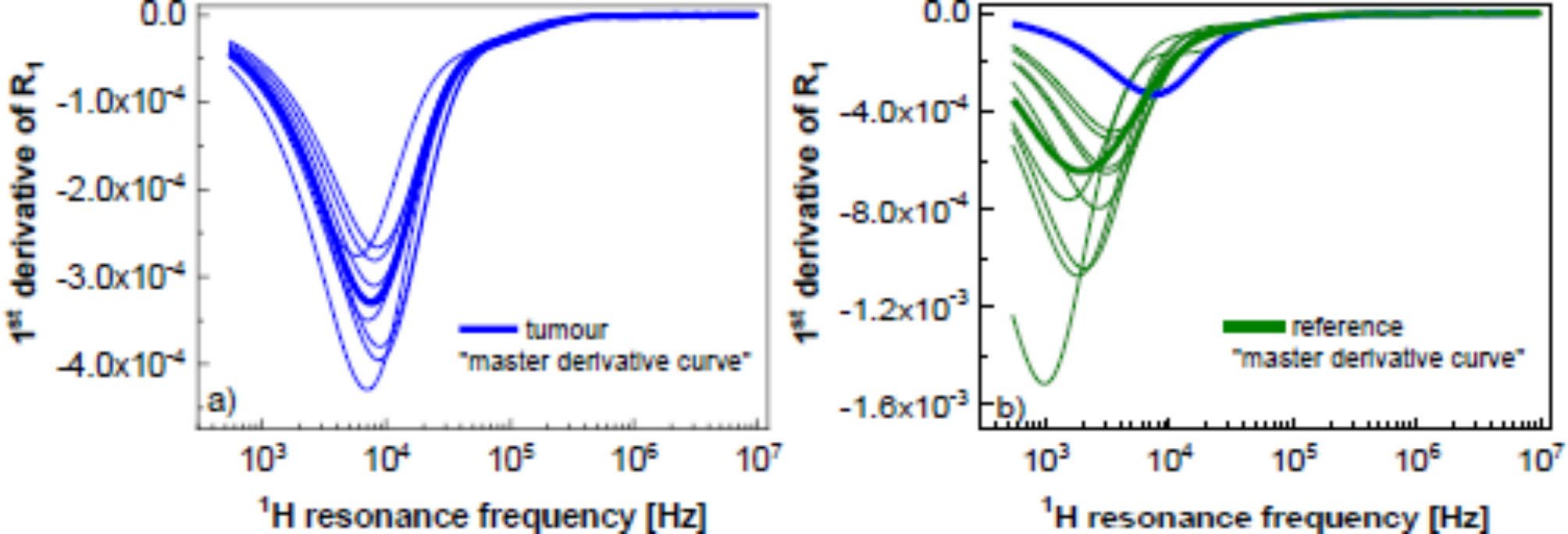
Derivatives of the relaxation rates over the resonance frequency for (**a**) pathological and (**b**) reference tissues. The master derivative curves (thick lines) being an average of the derivatives for the individual data sets are shown. (**b**) Includes the master derivative curve from (**a**) to enable a direct comparison (blue line). The derivatives have been obtained from the curves representing the fits in terms of Eq. 4.

The parametrisation can be exploited to reveal further differences between the relaxation data for the pathological and reference tissues. An interesting approach is to consider not the shape of the frequency dependencies of the spin-lattice relaxation rates, but their derivatives. Figure 3a and b show the derivatives of the relaxation data for the pathological and reference tissues, respectively. The derivatives have been averaged, creating a “master derivative curve” for both cases.

Both curves show a minimum as the relaxation rates decay with increasing frequency. In the case of pathological tissues, the minimum is less pronounced. The ratio between the amplitudes (depths) of the “master” derivative curves for the pathological and reference tissues yields 0.44 ± 0.03. The minimum of the derivative for the pathological tissues is shifted towards higher frequencies compared to the position of the minimum for the reference tissues. The averaged frequency position of the minimum for the pathological tissues yields 5.39 kHz ± 1.94 kHz, while for the reference tissues the averaged position of the minimum is 1.79 kHz ± 2.03 kHz.

In the Supplementary Appendix (Fig. A3) the comparison of the derivative curves for the pathological and reference tissues for each case is presented. The comparison shows that the two effects, i.e. a deeper minimum for the reference tissue and its position at a lower frequency compared to the pathological tissue, appear in all cases.

### Ratio between the relaxation rates for pathological and reference tissues

**Fig. 4:**
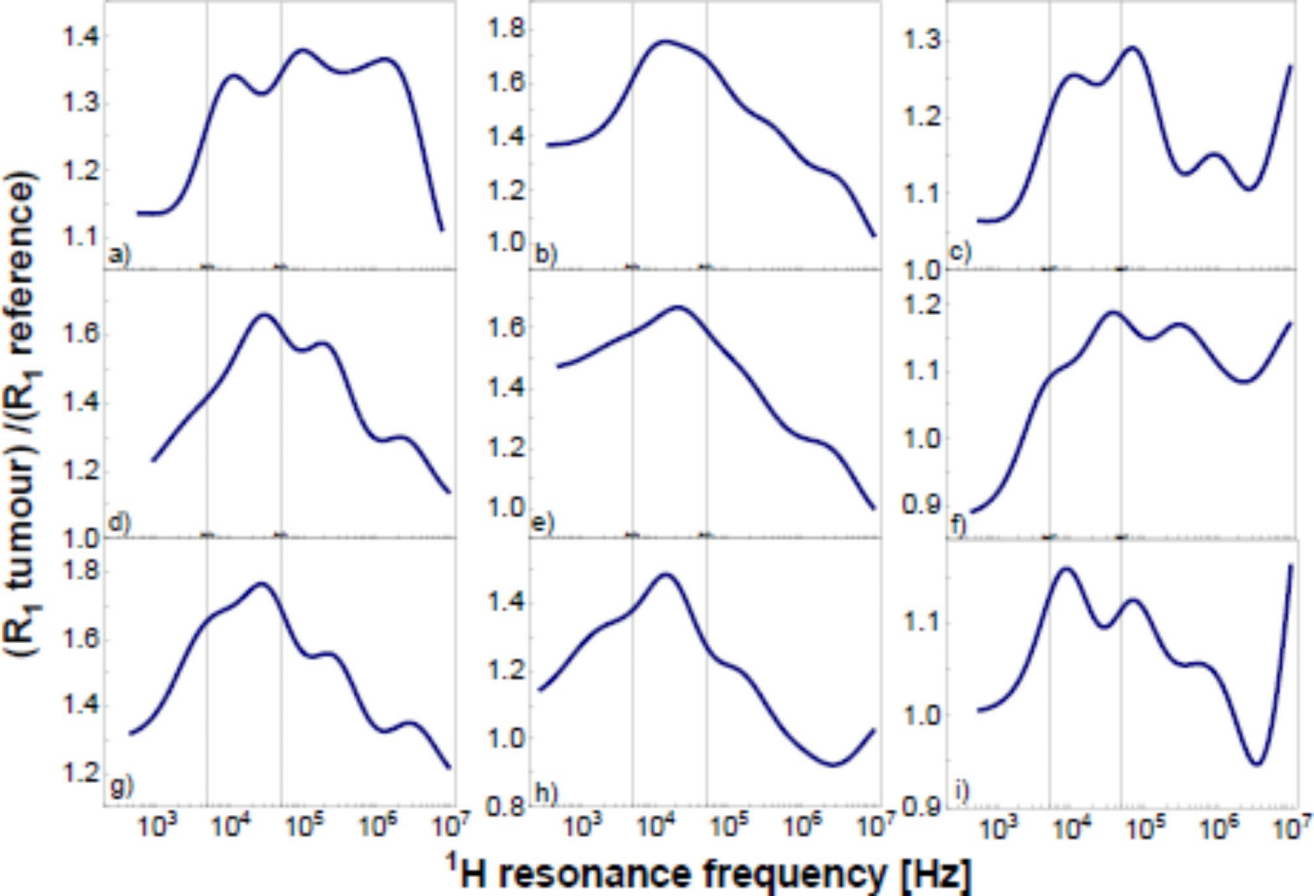
Ratio between ^1^H spin-lattice relaxation rates for pathological and reference tissues.

Although the main purpose of revealing characteristic relaxation markers for pathological tissues is to identify the pathological tissue not resorting to comparisons with other data sets, it is of interest to analyse the ratio between the relaxation rates for the pathological and reference tissues. The ratios are shown in Fig. 4 for the individual cases.

Although the shape of the frequency dependence of the ratio is quite complex, one can observe that in all cases there is a maximum in the frequency range from 5 kHz to 50 kHz.

### Scaling and grouping

**Fig. 5:**
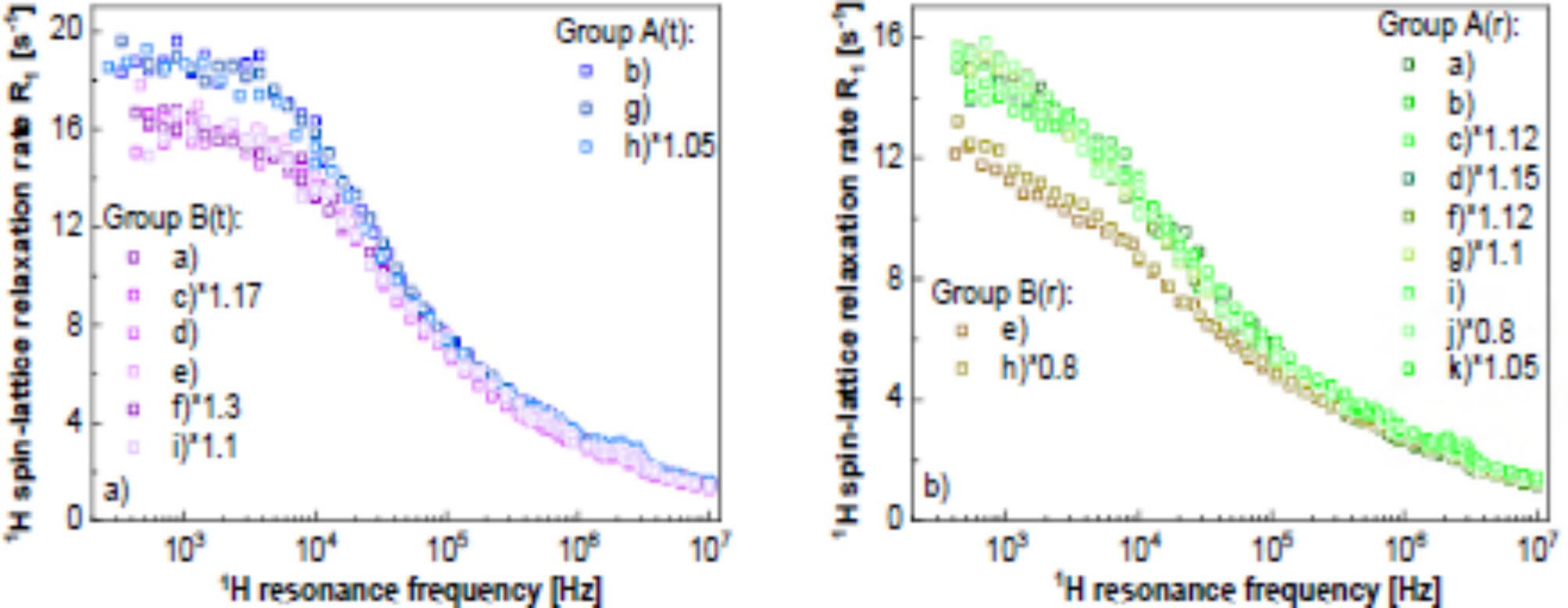
Grouped and scaled relaxation data for tumour (**a**) and reference, (**b**) tissues. The data were scaled by the factors given in the legend. A(t) and B(t) refer to group A and group B of the tumour tissues, while A(r) and B(r) refer to group A and group B of the reference tissues.

For the purpose of comparing the shapes of the frequency dependencies of the spin-lattice relaxation rates, not the arbitrary values of the relaxation rates, some of the data have been multiplied by a factor chosen in such a way so the data overlap in the high frequency limit. This concept has already been used in Fig. 1 to underline the differences in the shapes of the frequency dependencies of the relaxation rates for the pathological and reference tissues for the individual cases. Figure 5a shows the outcome of scaling the relaxation data for the pathological tissues, while Fig. 5b shows the analogous result for the reference tissues.

**Fig. 6:**
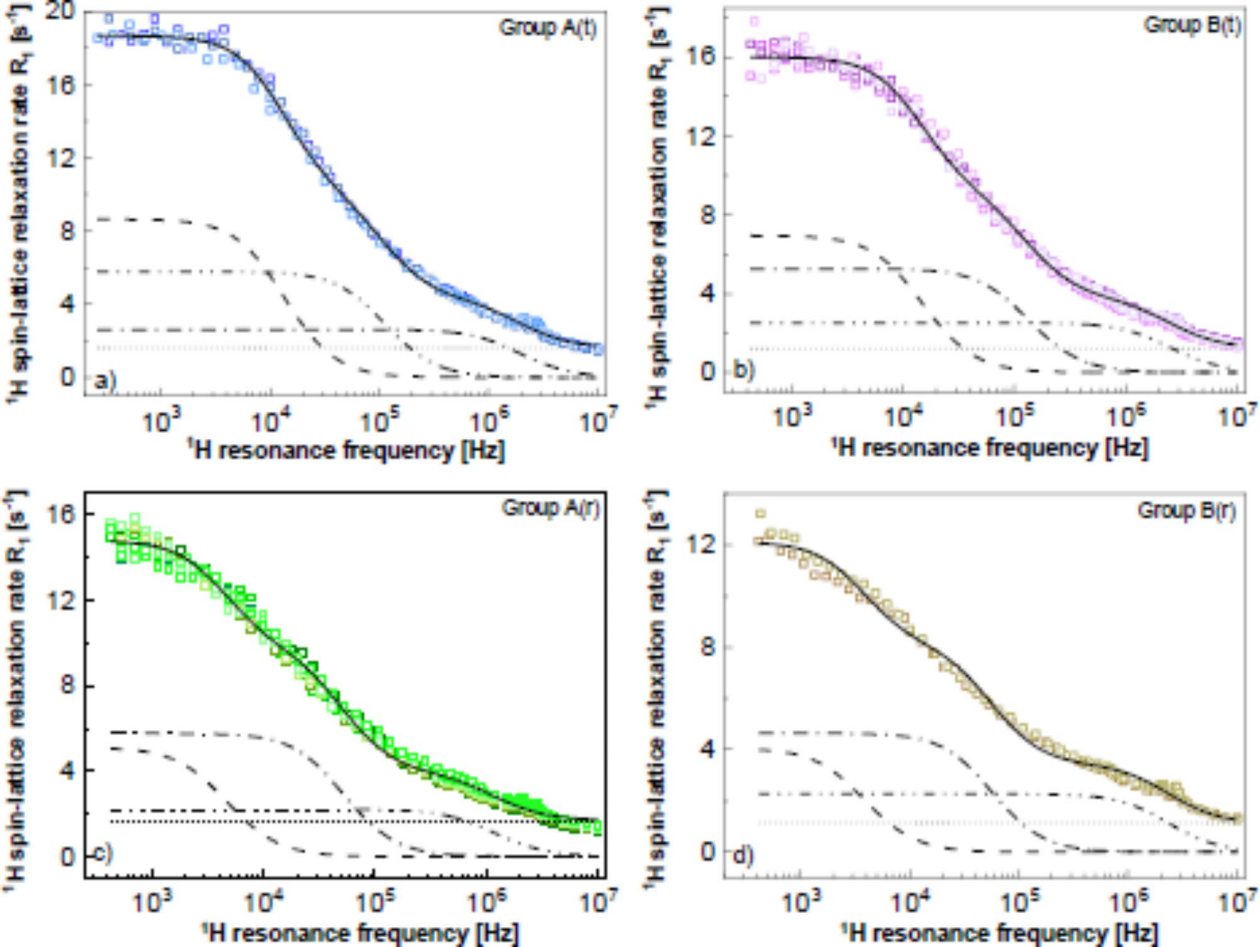
(**a–d**) Groups of ^1^H spin-lattice relaxation data reproduced in terms of Eq. 4 (solid lines). The fit has been decomposed into the relaxation contributions associated with slow (dashed lines), intermediate (dashed-dotted) and fast (dashed-dotted-dotted) lines; the frequency independent term, $\:A$, is represented by dotted lines.

For both the pathological and the reference tissues, one can distinguish two groups of data, but the groups are formed by different individual cases. Figure A4 (Supplementary Appendix) shows a comparison of the four groups. The data can be parametrised in terms of Eq. 4, as shown in Fig. 6a.

The obtained parameters are collected in Table 4.

**Table 4:** Parameters obtained from fitting the groups of relaxation data in terms of Eq. 4.

Group	$\:{C}_{s}$ [kHz^2^]	$\:{\tau\:}_{s}$ [ms]	$\:{C}_{i}$ [kHz^2^]	$\:{\tau\:}_{i}$ [ns]	$\:{C}_{f}$ [ kHz^2^]	$\:{\tau\:}_{f}$ [ns]	$\:A$ [s^−1^]
A(t)	0.261 ± 0.17	6.63 ± 0.26	1.45 ± 0.08	797 ± 56	11.2 ± 1.1	46.6 ± 3.9	1.63 ± 0.11
A(r)	0.0534 ± 0.0029	19.1 ± 0.8	0.675 ± 0.020	1710 ± 60	5.78 ± 3.28	76.5 ± 4.3	1.69 ± 0.04
B(t)	0.233 ± 0.013	5.97 ± 0.21	1.58 ± 0.62	667 ± 35	14.1 ± 2.8	35.8 ± 1.3	1.22± 0.11
B(r)	0.0346 ± 0.0036	23.4 ± 0.2	0.588 ± 0.314	1580 ± 104	12.1 ± 4.1	38.0 ± 1.8	1.12 ± 0.04

Following the approach presented in Sect. 3.1, it is of interest to compare the $\:\xi\:$ parameters obtained from the fitted curves in the frequency range from 1 to 10 kHz and from 10 kHz to 10 MHz; they yield: 20.1% for group 1 of tumour, 15.9% for group 2, 38.9% for group 1 of reference, 39.3% for group 2 of reference in the 1–10 kHz frequency range and 892.1% for group 1 of tumour, 790.8% for group 2, 502.3% for group 1 of reference, 568.5% for group 2 of reference in the 10 kHz-10 MHz frequency range.

In Fig. A5 (Supplementary Appendix), the derivative curves for the four groups are shown. The derivatives preserve the features described in Sect. 3.3 – the minimum is more pronounced for the reference tissues and shifted towards lower frequencies.

## Discussion

This work allowed us to develop some analytical methods to extract potential biomarkers of colorectal cancer, but at this stage any biological interpretation can only be hypothetical and further study is ongoing to clarify the origin of the signals observed.

Comparing ^1^H spin-lattice relaxation data for the pathological and reference colon tissues, one can make two observations. The first one is that the relaxation rates tend to coincide at the higher frequencies (above 1 MHz). The second observation is that in most cases (7 out of 9), the relaxation rates at low frequencies are larger for the pathological tissues than for the reference ones – at 1 kHz the averaged relaxation rates are 16.08 ± 2.11 and 14.36 ± 1.88 for the pathological and reference tissues, analogously at 10 kHz the values yield 13.31 ± 2.82 for the pathological tissues and 10.60 ± 1.36 for the reference ones. Although this finding is undoubtedly worth attention, in this work we aim to reveal relaxation factors characteristic of pathological and reference tissues that are not entirely based on the arbitrary values of the relaxation rates themselves, but on the shape of the frequency dependencies of the relaxation rates. The most straightforward factor to determine is the parameter $\:\xi\:$ reflecting the “steepness” of the relaxation rates versus the resonance frequency in a given frequency range. Even though the parameter $\:\xi\:$ calculated for the broad frequency range from 1 kHz to 10 MHz is different for the pathological and reference tissues (954.7 ± 145.5 for pathological and 695.8 ± 87.5 for reference), the most significant differences are observed in the low frequency range – the $\:\xi\:$ parameter corresponding to the frequency range from 1 kHz to 10 kHz yields 18.1 ± 4.5 for the pathological tissues and 34.8 ± 6.7 for the reference ones.

A quantitative description of the relaxation data has led to three pairs of parameters (the correlation time and the corresponding dipolar relaxation constant). As already pointed out, the most significant differences are revealed for the parameters characterising the slow dynamics: $\:{\tau\:}_{s}$ and $\:{C}_{s}$. This was to be expected as relaxation processes at low frequencies are associated with slow dynamics. The average dipolar relaxation constant, $\:{C}_{s}$, for the tumour tissues is larger by a factor of ~ 5 compared to that for the reference tissues, while the correlation time is shorter by a factor of ~ 4. The dipolar relaxation constant for the intermediate process, $\:{C}_{i}$, is larger by a factor of ~ 2 for the pathological tissues compared to the reference ones, while the correlation time, $\:{\tau\:}_{i}$, is shorter by a factor of ~ 2; the differences in the parameters $\:{C}_{f}\:$and $\:{\tau\:}_{f}$ are less significant.

To get a deeper insight into the characteristic shapes of the frequency dependencies of the relaxation rates, the derivative of the relaxation rates over the frequency range has been calculated. The minimum of the derivative corresponds to the frequency around which the changes in the relaxation rates are most significant. The absolute value of the minimum is by a factor of ~ 2 lower for the pathological tissues compared to the reference ones. As far as the frequency position of the minimum of the derivative is concerned, it is shifted towards higher frequencies for the pathological tissues (about 5 kHz) compared to the reference ones (about 2 kHz).

One can attempt to provide explanations for these effects: for instance, one could attribute the larger dipolar relaxation constant for the pathological tissues to a higher population of the fraction of water molecules strongly bound to the macromolecular matrix in the pathological tissues. The shorter correlation time associated with the tumour tissue also suggests that this matrix may be constituted of smaller proteins, or may be more loosely structured in tumours. This seems coherent with the histological analyses of tumour tissues, which are more disorganised than healthy ones.

Another possibility could be the exchange of water through cell membranes. Other groups have reported changes in NMRD profiles due to passive channels such aquaporins^10,11,54^, which modulate the intracellular contribution to the NMRD profile. Active channels are unlikely to contribute in fixed tissues, but membrane damage may provide additional passive channels.

The presence of two subsets of NMRD profiles indicates additional relaxation mechanisms at fields below 2 mT. Determining the exact origin would require detailed analysis of the tissue components, which was not planned for this work. One possibility could be Further studies are under way using fresh tissue samples to better understand the biological pathways relating to our findings.

## Conclusions

Several quantities have been indicated as possible markers of pathological changes in tissues reflected by ^1^H spin-lattice NMR relaxometry. The quantities have been obtained and compared for colon tissues. It has been shown that the relative changes in the relaxation rates in the low frequency range differ significantly, depending on the state of the tissue – the changes are smaller for pathological tissues compared to the reference. Analogously, a minimum of the first derivative of the relaxation rate over the resonance frequency has been revealed. The minimum is more pronounced for the reference tissues and shifted towards lower frequencies compared to the pathological ones. A quantitative parametrisation of the relaxation data in terms of Lorentzian spectral densities has led to a set of dipolar relaxation constants and associated correlation times that can be used for the purpose of differentiating between the state of the tissue. The ^1^H spin-lattice relaxation data for the pathological and reference tissues tend to coincide at higher frequencies, while at lower frequencies the differences are clearly pronounced. The relaxation data for the pathological and reference tissues, after a re-scaling (multiplying by a frequency independent factor), can be attributed to two groups (for each case) that likely reflect their different structural properties. At this stage it is difficult to determine the biological origin of these signals and further work is ongoing using fresh colorectal cancer tissues to validate our findings for in vivo applications.

## Methods

### Tissue samples

Twelve formalin-fixed colorectal tissue samples were obtained from colorectal cancer patients during surgery. The extraction process was specifically aimed at obtaining tissue cores from two different regions as follows: tumour area (*n* = 12) and non-involved healthy tissue (*n* = 12). R1 measurements were acquired after pathology examination so as not to interfere with normal patient care. These samples were obtained fixed in a 4% solution of formaldehyde. Some samples were excluded due to technical measurement errors (Healthy *n* = 3, Tumour *n* = 3). Ethical approval was granted by the Grampian Biorepository Scientific Access Group (tissue requests TR000068) prior to commencing this prospective study, and the patients’ informed consents were obtained from all participants. All methods were performed in accordance with the Good Clinical Practices for Clinical Trials UK. Confidentiality and privacy of the participants data were maintained throughout the research process.

### FFC-NMR acquisitions

In this study, we used a commercial benchtop FFC NMR relaxometer (SMARtracer; Stelar S.r.l., Mede, Italy). Tissue cores around 1 ml in volume were placed into flat-bottom tubes (Scientific Glass Laboratories Ltd, Tunstall, UK, G050/18 model) with a diameter of 10 mm and a length of 50 mm and analysed at a controlled temperature of 37 °C± 0.1 °C. R1 dispersion profiles were analysed in the fields lying between 1 kHz and 10 MHz proton Larmor frequency (PLF) range (corresponding to applied magnetic field B0 = 0.02 mT to 0.2 T). The overall acquisition time of the NMR dispersion (NMRD) profile was approximately 20 min per sample. The 1 H spin-lattice relaxation rate of these samples was investigated using a saturation recovery pulse sequence.

This work received support from the EURELAX COST Action CA15209, supported by COST (European Cooperation in Science and Technology).

## Electronic supplementary material

Below is the link to the electronic supplementary material.

Supplementary Material 1

## Data Availability

The data that support the findings of this study are not openly available due to reasons of sensitivity and are available from the corresponding author upon reasonable request. Data are located in controlled access data storage at the University of Aberdeen.
