# Supplementary material for: Markers of low field NMR relaxation features of tissues
Source: Sci Rep. 2024 Oct 22;14:24901. doi: 10.1038/s41598-024-74055-7 (PMC11496659; doi:10.1038/s41598-024-74055-7)
Supplement: Supplementary file 1 — Supplementary Material 1 [file 41598_2024_74055_MOESM1_ESM.docx]

**Appendix**

Figure A1.^1^H spin-lattice relaxation rates for pathological and reference (background) colon tissues. Solid lines – splines of the data. Vertical lines indicate the frequency range used for calculating the $\xi$ parameter.

Figure A2. ^1^H spin-lattice relaxation rates for reference colon tissues reproduce in terms of Eq.4 (solid lines). The fit has been decomposed into the relaxation contributions associated with slow (dashed lines), intermediate (dashed-dotted) and fast (dashed-dotted-dotted) lines; the frequency independent term, $A$, is represented by dotted lines.

Figure A3**.** Comparison of derivatives of ^1^H spin-lattice relaxation rates over the resonance frequency for pathological and reference tissues. The derivatives have been obtained from the curves representing the fits in terms of Eq.4.

Figure A4. Comparison of four groups of relaxation data.

Figure A5. Comparison of derivatives of ^1^H spin-lattice relaxation rates over the resonance frequency for groups of pathological and reference tissues. The derivatives have been obtained from the curves representing the fits in terms of Eq.4.
